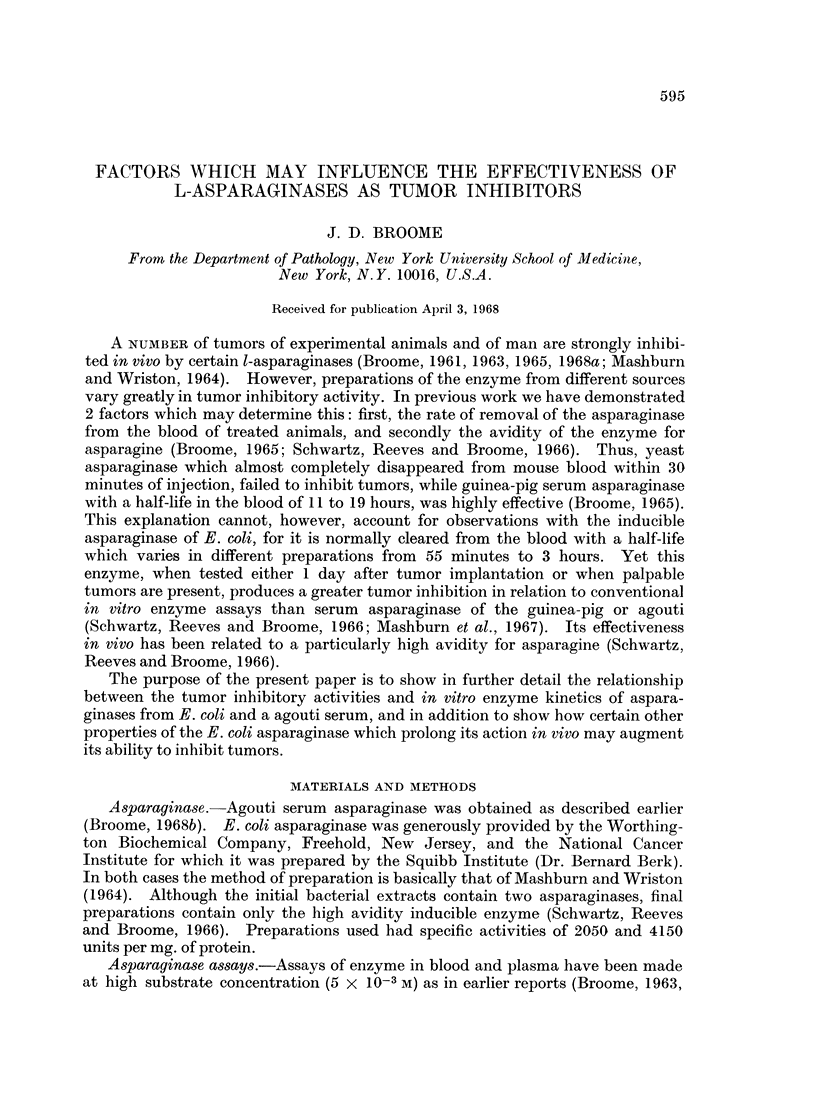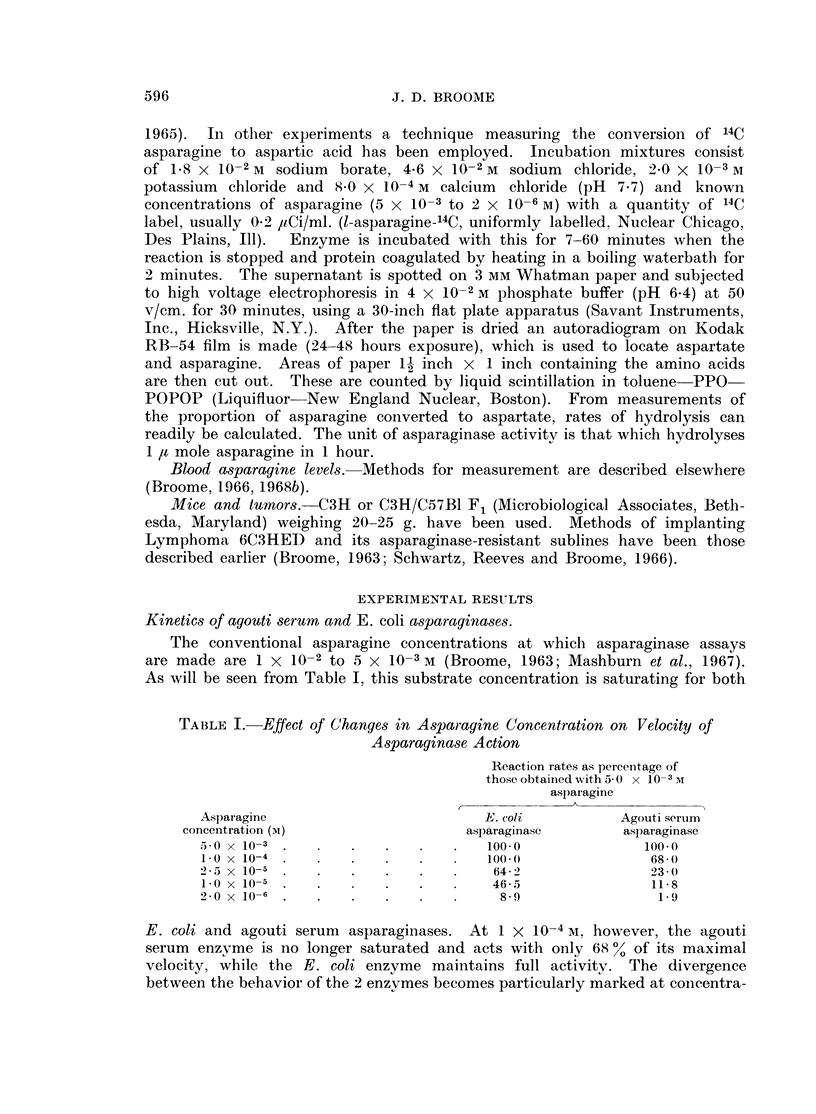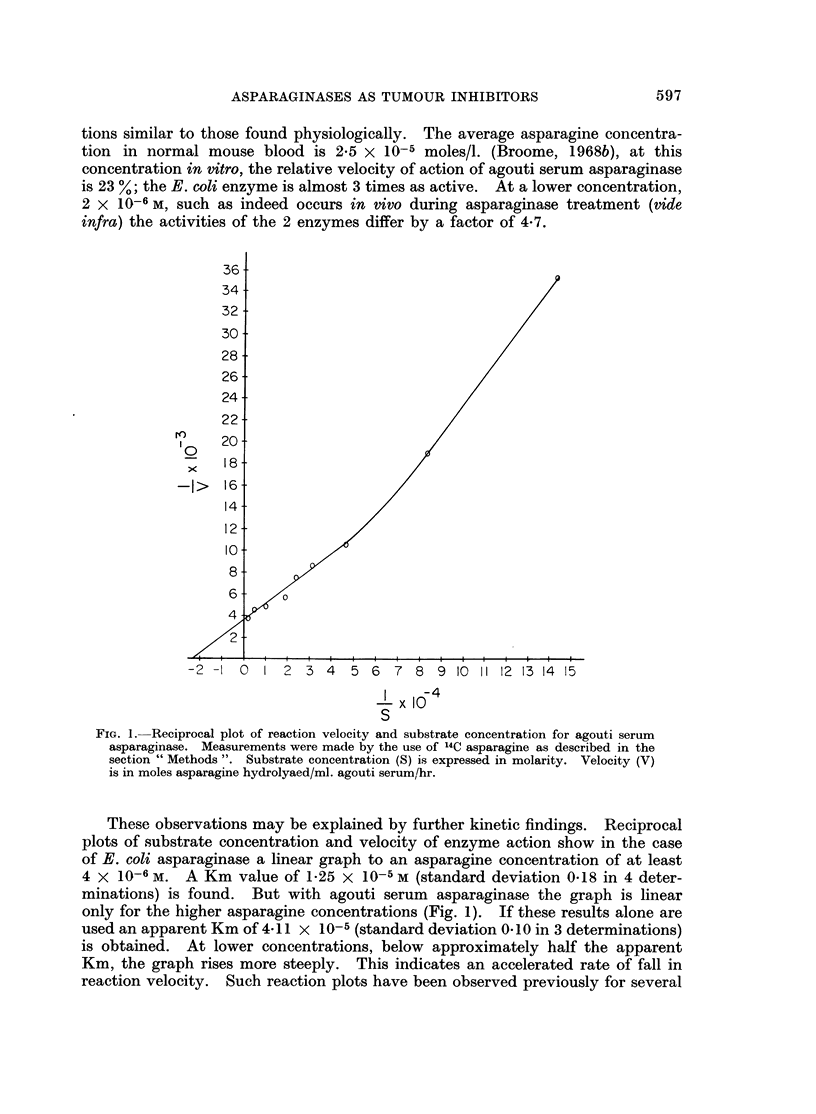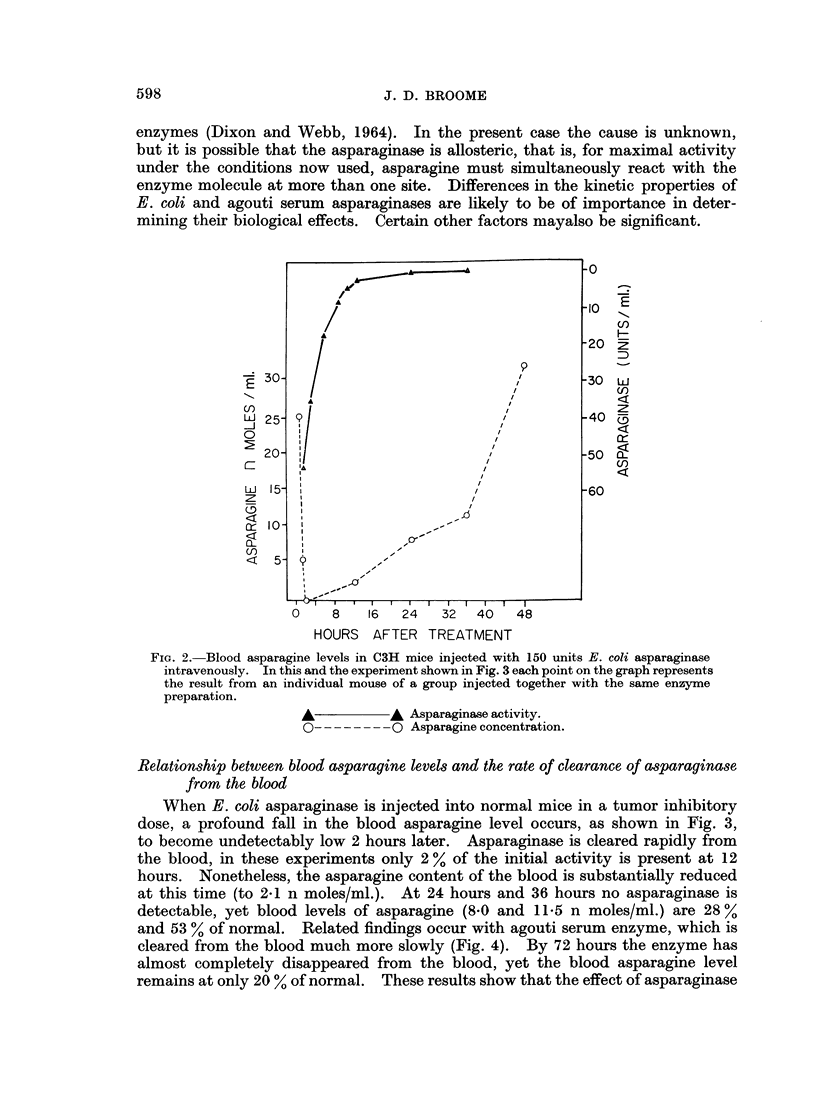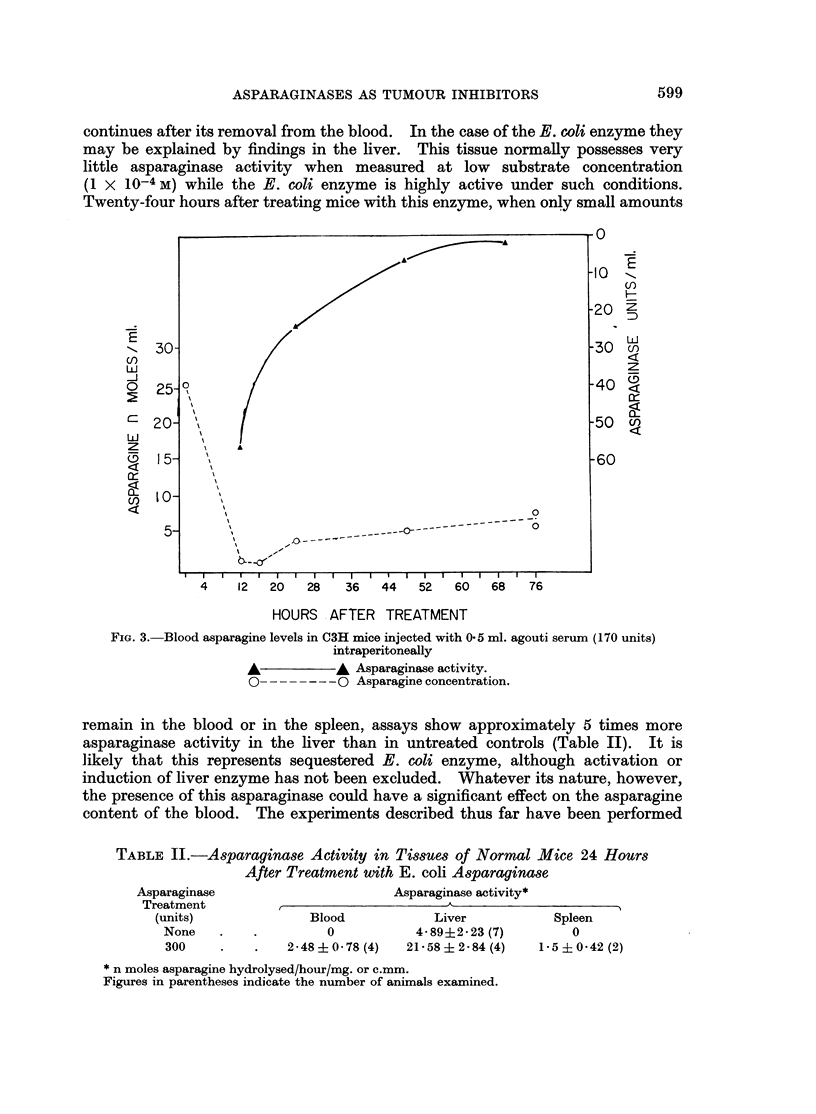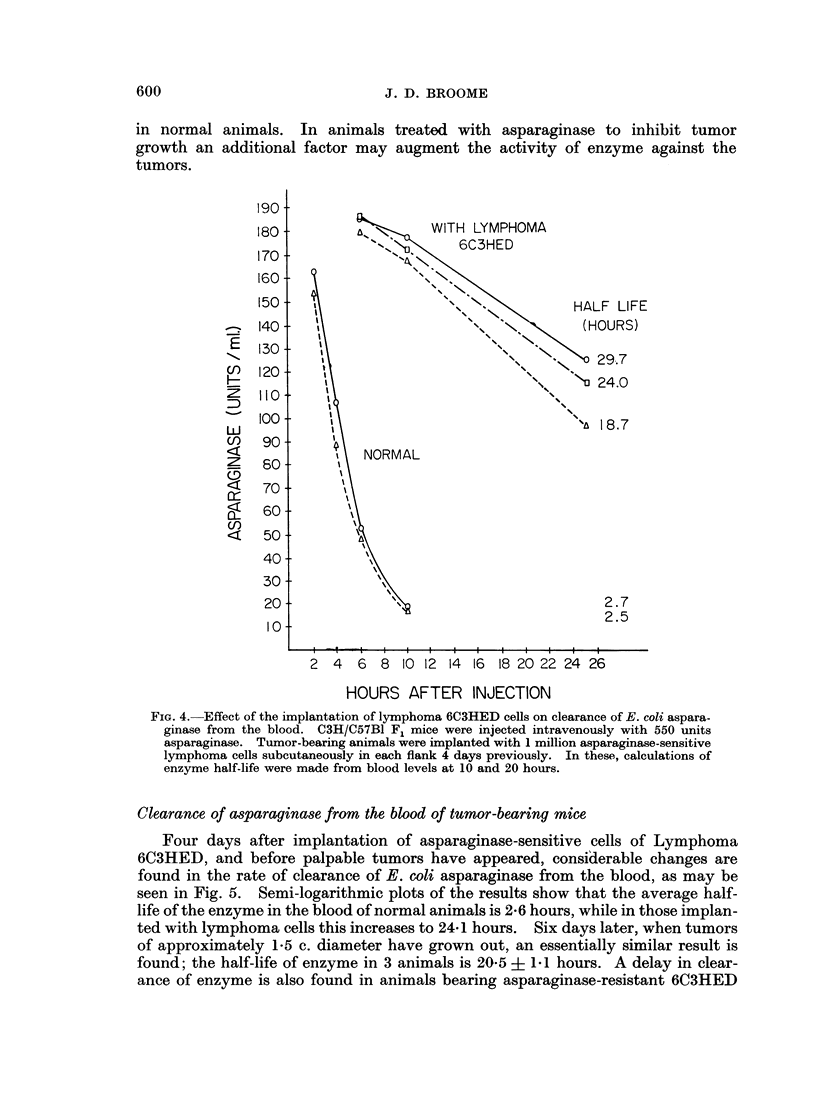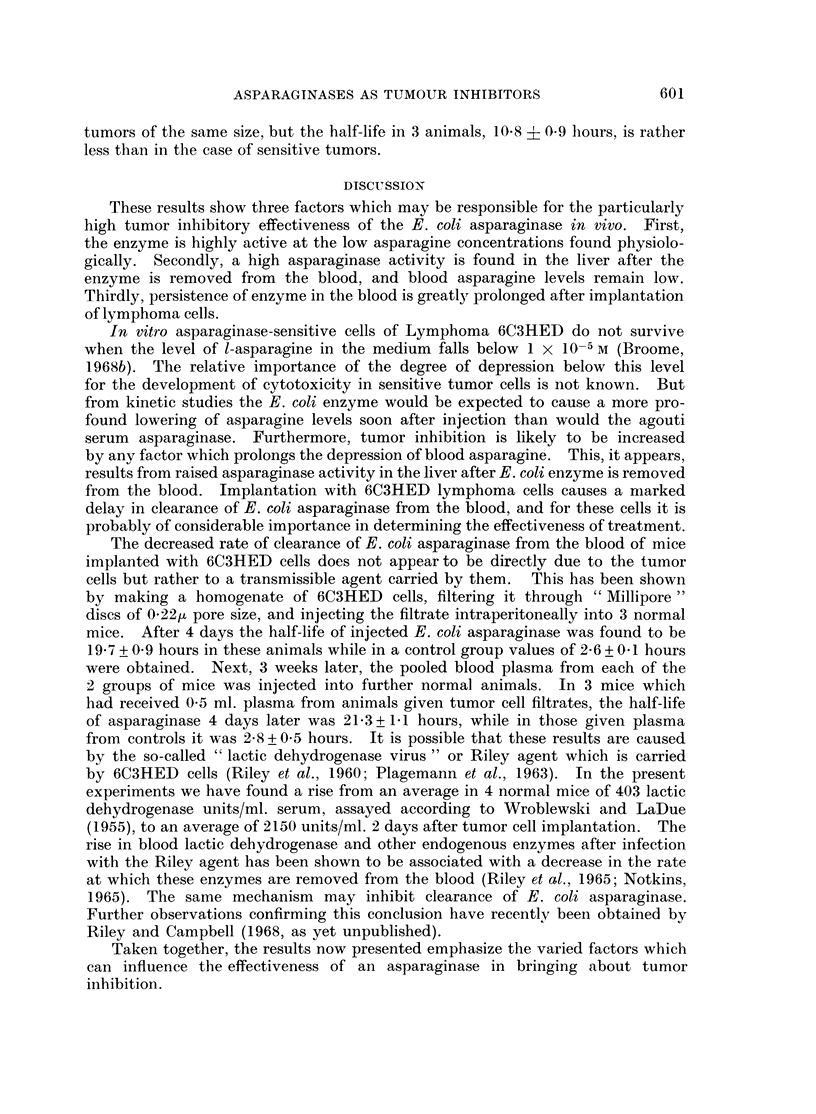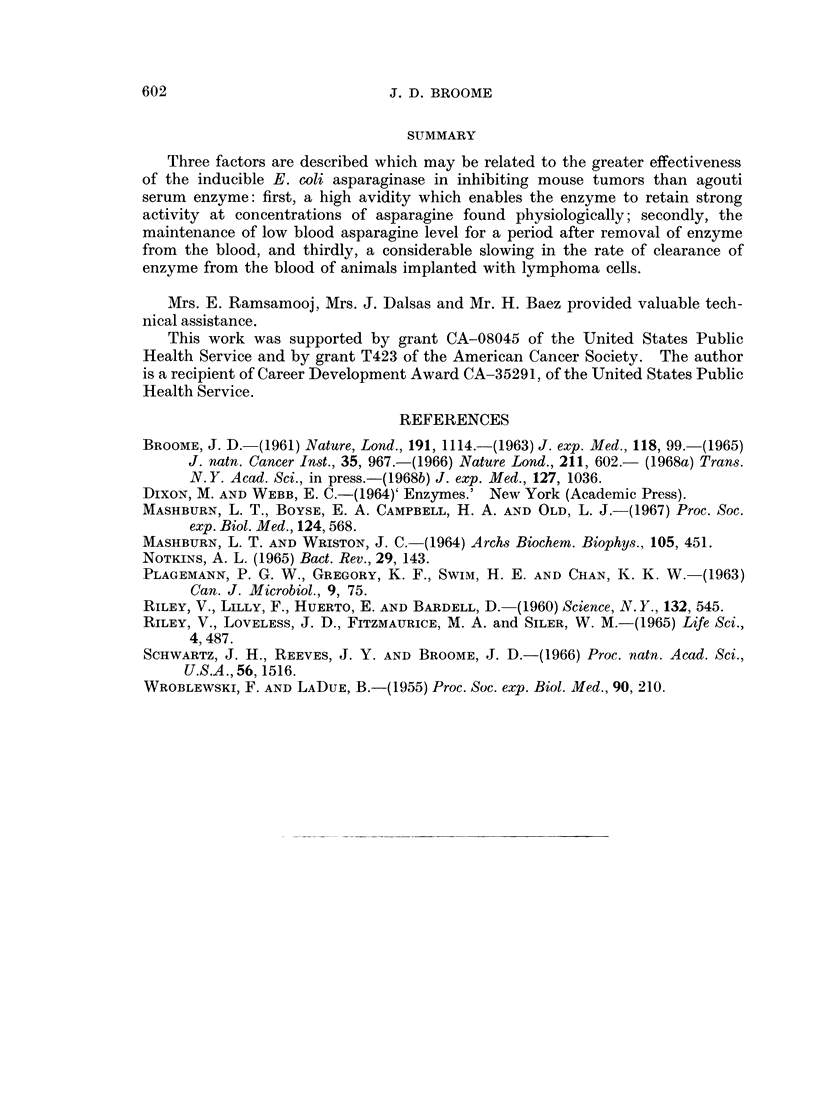# Factors which may influence the effectiveness of L-asparaginases as tumor inhibitors.

**DOI:** 10.1038/bjc.1968.71

**Published:** 1968-09

**Authors:** J. D. Broome


### FACTORS WHICH MAY INFLUENCE THE EFFECTIVENESS OF L-ASPARAGINASES AS TUMOR INHIBITORS

J. D. BROOME

*From the Department of Pathology, New York University School of Medicine, New York, N.Y. 10016, U.S.A.*

Received for publication April 3, 1968

A NUMBER of tumors of experimental animals and of man are strongly inhibited *in vivo* by certain *l*-asparaginases (Broome, 1961, 1963, 1965, 1968*a*; Mashburn and Wriston, 1964). However, preparations of the enzyme from different sources vary greatly in tumor inhibitory activity. In previous work we have demonstrated 2 factors which may determine this: first, the rate of removal of the asparaginase from the blood of treated animals, and secondly the avidity of the enzyme for asparagine (Broome, 1965; Schwartz, Reeves and Broome, 1966). Thus, yeast asparaginase which almost completely disappeared from mouse blood within 30 minutes of injection, failed to inhibit tumors, while guinea-pig serum asparaginase with a half-life in the blood of 11 to 19 hours, was highly effective (Broome, 1965). This explanation cannot, however, account for observations with the inducible asparaginase of *E. coli*, for it is normally cleared from the blood with a half-life which varies in different preparations from 55 minutes to 3 hours. Yet this enzyme, when tested either 1 day after tumor implantation or when palpable tumors are present, produces a greater tumor inhibition in relation to conventional *in vitro* enzyme assays than serum asparaginase of the guinea-pig or agouti (Schwartz, Reeves and Broome, 1966; Mashburn *et al.*, 1967). Its effectiveness *in vivo* has been related to a particularly high avidity for asparagine (Schwartz, Reeves and Broome, 1966).

The purpose of the present paper is to show in further detail the relationship between the tumor inhibitory activities and *in vitro* enzyme kinetics of asparaginases from *E. coli* and a agouti serum, and in addition to show how certain other properties of the *E. coli* asparaginase which prolong its action *in vivo* may augment its ability to inhibit tumors.

### MATERIALS AND METHODS

*Asparaginase.*—Agouti serum asparaginase was obtained as described earlier (Broome, 1968*b*). *E. coli* asparaginase was generously provided by the Worthington Biochemical Company, Freehold, New Jersey, and the National Cancer Institute for which it was prepared by the Squibb Institute (Dr. Bernard Berk). In both cases the method of preparation is basically that of Mashburn and Wriston (1964). Although the initial bacterial extracts contain two asparaginases, final preparations contain only the high avidity inducible enzyme (Schwartz, Reeves and Broome, 1966). Preparations used had specific activities of 2050 and 4150 units per mg. of protein.

*Asparaginase assays.*—Assays of enzyme in blood and plasma have been made at high substrate concentration ($5 \times 10^{-3}$ M) as in earlier reports (Broome, 1963,

1965). In other experiments a technique measuring the conversion of $^{14}C$ asparagine to aspartic acid has been employed. Incubation mixtures consist of $1 \cdot 8 \times 10^{-2}$ M sodium borate, $4 \cdot 6 \times 10^{-2}$ M sodium chloride, $2 \cdot 0 \times 10^{-3}$ M potassium chloride and $8 \cdot 0 \times 10^{-4}$ M calcium chloride (pH 7·7) and known concentrations of asparagine ($5 \times 10^{-3}$ to $2 \times 10^{-6}$ M) with a quantity of $^{14}C$ label, usually 0·2 $\mu$Ci/ml. (*l*-asparagine-$^{14}C$, uniformly labelled, Nuclear Chicago, Des Plains, Ill). Enzyme is incubated with this for 7–60 minutes when the reaction is stopped and protein coagulated by heating in a boiling waterbath for 2 minutes. The supernatant is spotted on 3 MM Whatman paper and subjected to high voltage electrophoresis in $4 \times 10^{-2}$ M phosphate buffer (pH 6·4) at 50 v/cm. for 30 minutes, using a 30-inch flat plate apparatus (Savant Instruments, Inc., Hicksville, N.Y.). After the paper is dried an autoradiogram on Kodak RB–54 film is made (24–48 hours exposure), which is used to locate aspartate and asparagine. Areas of paper $1\frac{1}{2}$ inch × 1 inch containing the amino acids are then cut out. These are counted by liquid scintillation in toluene—PPO—POPOP (Liquifluor—New England Nuclear, Boston). From measurements of the proportion of asparagine converted to aspartate, rates of hydrolysis can readily be calculated. The unit of asparaginase activity is that which hydrolyses 1 $\mu$ mole asparagine in 1 hour.

*Blood asparagine levels.*—Methods for measurement are described elsewhere (Broome, 1966, 1968*b*).

*Mice and tumors.*—C3H or C3H/C57Bl $F_1$ (Microbiological Associates, Bethesda, Maryland) weighing 20–25 g. have been used. Methods of implanting Lymphoma 6C3HED and its asparaginase-resistant sublines have been those described earlier (Broome, 1963; Schwartz, Reeves and Broome, 1966).

### EXPERIMENTAL RESULTS

### *Kinetics of agouti serum and* E. coli *asparaginases.*

The conventional asparagine concentrations at which asparaginase assays are made are $1 \times 10^{-2}$ to $5 \times 10^{-3}$ M (Broome, 1963; Mashburn *et al.*, 1967). As will be seen from Table I, this substrate concentration is saturating for both

TABLE I.—*Effect of Changes in Asparagine Concentration on Velocity of Asparaginase Action*

| Asparagine concentration (M) | Reaction rates as percentage of those obtained with $5 \cdot 0 \times 10^{-3}$ M asparagine: *E. coli* asparaginase | Agouti serum asparaginase |
|---|---|---|
| $5 \cdot 0 \times 10^{-3}$ | 100·0 | 100·0 |
| $1 \cdot 0 \times 10^{-4}$ | 100·0 | 68·0 |
| $2 \cdot 5 \times 10^{-5}$ | 64·2 | 23·0 |
| $1 \cdot 0 \times 10^{-5}$ | 46·5 | 11·8 |
| $2 \cdot 0 \times 10^{-6}$ | 8·9 | 1·9 |

*E. coli* and agouti serum asparaginases. At $1 \times 10^{-4}$ M, however, the agouti serum enzyme is no longer saturated and acts with only 68% of its maximal velocity, while the *E. coli* enzyme maintains full activity. The divergence between the behavior of the 2 enzymes becomes particularly marked at concentrations similar to those found physiologically. The average asparagine concentration in normal mouse blood is $2 \cdot 5 \times 10^{-5}$ moles/l. (Broome, 1968*b*), at this concentration *in vitro*, the relative velocity of action of agouti serum asparaginase is 23 %; the *E. coli* enzyme is almost 3 times as active. At a lower concentration, $2 \times 10^{-6}$ M, such as indeed occurs *in vivo* during asparaginase treatment (*vide infra*) the activities of the 2 enzymes differ by a factor of 4·7.

FIG. 1.—Reciprocal plot of reaction velocity and substrate concentration for agouti serum asparaginase. Measurements were made by the use of $^{14}C$ asparagine as described in the section " Methods ". Substrate concentration (S) is expressed in molarity. Velocity (V) is in moles asparagine hydrolyaed/ml. agouti serum/hr.

These observations may be explained by further kinetic findings. Reciprocal plots of substrate concentration and velocity of enzyme action show in the case of *E. coli* asparaginase a linear graph to an asparagine concentration of at least $4 \times 10^{-6}$ M. A Km value of $1 \cdot 25 \times 10^{-5}$ M (standard deviation 0·18 in 4 determinations) is found. But with agouti serum asparaginase the graph is linear only for the higher asparagine concentrations (Fig. 1). If these results alone are used an apparent Km of $4 \cdot 11 \times 10^{-5}$ (standard deviation 0·10 in 3 determinations) is obtained. At lower concentrations, below approximately half the apparent Km, the graph rises more steeply. This indicates an accelerated rate of fall in reaction velocity. Such reaction plots have been observed previously for several enzymes (Dixon and Webb, 1964). In the present case the cause is unknown, but it is possible that the asparaginase is allosteric, that is, for maximal activity under the conditions now used, asparagine must simultaneously react with the enzyme molecule at more than one site. Differences in the kinetic properties of *E. coli* and agouti serum asparaginases are likely to be of importance in determining their biological effects. Certain other factors mayalso be significant.

FIG. 2.—Blood asparagine levels in C3H mice injected with 150 units *E. coli* asparaginase intravenously. In this and the experiment shown in Fig. 3 each point on the graph represents the result from an individual mouse of a group injected together with the same enzyme preparation.

▲———▲ Asparaginase activity.
○- - - -○ Asparagine concentration.

*Relationship between blood asparagine levels and the rate of clearance of asparaginase from the blood*

When *E. coli* asparaginase is injected into normal mice in a tumor inhibitory dose, a profound fall in the blood asparagine level occurs, as shown in Fig. 3, to become undetectably low 2 hours later. Asparaginase is cleared rapidly from the blood, in these experiments only 2 % of the initial activity is present at 12 hours. Nonetheless, the asparagine content of the blood is substantially reduced at this time (to 2·1 n moles/ml.). At 24 hours and 36 hours no asparaginase is detectable, yet blood levels of asparagine (8·0 and 11·5 n moles/ml.) are 28 % and 53 % of normal. Related findings occur with agouti serum enzyme, which is cleared from the blood much more slowly (Fig. 4). By 72 hours the enzyme has almost completely disappeared from the blood, yet the blood asparagine level remains at only 20 % of normal. These results show that the effect of asparaginase continues after its removal from the blood. In the case of the *E. coli* enzyme they may be explained by findings in the liver. This tissue normally possesses very little asparaginase activity when measured at low substrate concentration ($1 \times 10^{-4}$ M) while the *E. coli* enzyme is highly active under such conditions. Twenty-four hours after treating mice with this enzyme, when only small amounts

Fig. 3.—Blood asparagine levels in C3H mice injected with 0·5 ml. agouti serum (170 units) intraperitoneally

▲——————▲ Asparaginase activity.
○– – – – – – – –○ Asparagine concentration.

remain in the blood or in the spleen, assays show approximately 5 times more asparaginase activity in the liver than in untreated controls (Table II). It is likely that this represents sequestered *E. coli* enzyme, although activation or induction of liver enzyme has not been excluded. Whatever its nature, however, the presence of this asparaginase could have a significant effect on the asparagine content of the blood. The experiments described thus far have been performed

Table II.—*Asparaginase Activity in Tissues of Normal Mice* 24 *Hours After Treatment with* E. coli *Asparaginase*

| Asparaginase Treatment (units) | Asparaginase activity* | | |
|---|---|---|---|
| | Blood | Liver | Spleen |
| None . . | 0 | 4·89±2·23 (7) | 0 |
| 300 . . | 2·48 ± 0·78 (4) | 21·58 ± 2·84 (4) | 1·5 ± 0·42 (2) |

* n moles asparagine hydrolysed/hour/mg. or c.mm.
Figures in parentheses indicate the number of animals examined.

in normal animals. In animals treated with asparaginase to inhibit tumor growth an additional factor may augment the activity of enzyme against the tumors.

FIG. 4.—Effect of the implantation of lymphoma 6C3HED cells on clearance of *E. coli* asparaginase from the blood. C3H/C57Bl $F_1$ mice were injected intravenously with 550 units asparaginase. Tumor-bearing animals were implanted with 1 million asparaginase-sensitive lymphoma cells subcutaneously in each flank 4 days previously. In these, calculations of enzyme half-life were made from blood levels at 10 and 20 hours.

*Clearance of asparaginase from the blood of tumor-bearing mice*

Four days after implantation of asparaginase-sensitive cells of Lymphoma 6C3HED, and before palpable tumors have appeared, considerable changes are found in the rate of clearance of *E. coli* asparaginase from the blood, as may be seen in Fig. 5. Semi-logarithmic plots of the results show that the average half-life of the enzyme in the blood of normal animals is 2·6 hours, while in those implanted with lymphoma cells this increases to 24·1 hours. Six days later, when tumors of approximately 1·5 c. diameter have grown out, an essentially similar result is found; the half-life of enzyme in 3 animals is 20·5 ± 1·1 hours. A delay in clearance of enzyme is also found in animals bearing asparaginase-resistant 6C3HED

tumors of the same size, but the half-life in 3 animals, $10 \cdot 8 \pm 0 \cdot 9$ hours, is rather less than in the case of sensitive tumors.

### DISCUSSION

These results show three factors which may be responsible for the particularly high tumor inhibitory effectiveness of the *E. coli* asparaginase *in vivo.* First, the enzyme is highly active at the low asparagine concentrations found physiologically. Secondly, a high asparaginase activity is found in the liver after the enzyme is removed from the blood, and blood asparagine levels remain low. Thirdly, persistence of enzyme in the blood is greatly prolonged after implantation of lymphoma cells.

*In vitro* asparaginase-sensitive cells of Lymphoma 6C3HED do not survive when the level of *l*-asparagine in the medium falls below $1 \times 10^{-5}$ M (Broome, 1968*b*). The relative importance of the degree of depression below this level for the development of cytotoxicity in sensitive tumor cells is not known. But from kinetic studies the *E. coli* enzyme would be expected to cause a more profound lowering of asparagine levels soon after injection than would the agouti serum asparaginase. Furthermore, tumor inhibition is likely to be increased by any factor which prolongs the depression of blood asparagine. This, it appears, results from raised asparaginase activity in the liver after *E. coli* enzyme is removed from the blood. Implantation with 6C3HED lymphoma cells causes a marked delay in clearance of *E. coli* asparaginase from the blood, and for these cells it is probably of considerable importance in determining the effectiveness of treatment.

The decreased rate of clearance of *E. coli* asparaginase from the blood of mice implanted with 6C3HED cells does not appear to be directly due to the tumor cells but rather to a transmissible agent carried by them. This has been shown by making a homogenate of 6C3HED cells, filtering it through " Millipore " discs of $0 \cdot 22\mu$ pore size, and injecting the filtrate intraperitoneally into 3 normal mice. After 4 days the half-life of injected *E. coli* asparaginase was found to be $19 \cdot 7 \pm 0 \cdot 9$ hours in these animals while in a control group values of $2 \cdot 6 \pm 0 \cdot 1$ hours were obtained. Next, 3 weeks later, the pooled blood plasma from each of the 2 groups of mice was injected into further normal animals. In 3 mice which had received 0·5 ml. plasma from animals given tumor cell filtrates, the half-life of asparaginase 4 days later was $21 \cdot 3 \pm 1 \cdot 1$ hours, while in those given plasma from controls it was $2 \cdot 8 \pm 0 \cdot 5$ hours. It is possible that these results are caused by the so-called " lactic dehydrogenase virus " or Riley agent which is carried by 6C3HED cells (Riley *et al.*, 1960; Plagemann *et al.*, 1963). In the present experiments we have found a rise from an average in 4 normal mice of 403 lactic dehydrogenase units/ml. serum, assayed according to Wroblewski and LaDue (1955), to an average of 2150 units/ml. 2 days after tumor cell implantation. The rise in blood lactic dehydrogenase and other endogenous enzymes after infection with the Riley agent has been shown to be associated with a decrease in the rate at which these enzymes are removed from the blood (Riley *et al.*, 1965; Notkins, 1965). The same mechanism may inhibit clearance of *E. coli* asparaginase. Further observations confirming this conclusion have recently been obtained by Riley and Campbell (1968, as yet unpublished).

Taken together, the results now presented emphasize the varied factors which can influence the effectiveness of an asparaginase in bringing about tumor inhibition.

SUMMARY

Three factors are described which may be related to the greater effectiveness of the inducible *E. coli* asparaginase in inhibiting mouse tumors than agouti serum enzyme: first, a high avidity which enables the enzyme to retain strong activity at concentrations of asparagine found physiologically; secondly, the maintenance of low blood asparagine level for a period after removal of enzyme from the blood, and thirdly, a considerable slowing in the rate of clearance of enzyme from the blood of animals implanted with lymphoma cells.

Mrs. E. Ramsamooj, Mrs. J. Dalsas and Mr. H. Baez provided valuable technical assistance.

This work was supported by grant CA–08045 of the United States Public Health Service and by grant T423 of the American Cancer Society. The author is a recipient of Career Development Award CA–35291, of the United States Public Health Service.

### REFERENCES

BROOME, J. D.—(1961) *Nature, Lond.*, **191**, 1114.—(1963) *J. exp. Med.*, **118**, 99.—(1965) *J. natn. Cancer Inst.*, **35**, 967.—(1966) *Nature Lond.*, **211**, 602.— (1968*a*) *Trans. N.Y. Acad. Sci.*, in press.—(1968*b*) *J. exp. Med.*, **127**, 1036.

DIXON, M. AND WEBB, E. C.—(1964)' Enzymes.' New York (Academic Press).

MASHBURN, L. T., BOYSE, E. A. CAMPBELL, H. A. AND OLD, L. J.—(1967) *Proc. Soc. exp. Biol. Med.*, **124**, 568.

MASHBURN, L. T. AND WRISTON, J. C.—(1964) *Archs Biochem. Biophys.*, **105**, 451.

NOTKINS, A. L. (1965) *Bact. Rev.*, **29**, 143.

PLAGEMANN, P. G. W., GREGORY, K. F., SWIM, H. E. AND CHAN, K. K. W.—(1963) *Can. J. Microbiol.*, **9**, 75.

RILEY, V., LILLY, F., HUERTO, E. AND BARDELL, D.—(1960) *Science, N.Y.*, **132**, 545.

RILEY, V., LOVELESS, J. D., FITZMAURICE, M. A. and SILER, W. M.—(1965) *Life Sci.*, **4**, 487.

SCHWARTZ, J. H., REEVES, J. Y. AND BROOME, J. D.—(1966) *Proc. natn. Acad. Sci., U.S.A.*, **56**, 1516.

WROBLEWSKI, F. AND LADUE, B.—(1955) *Proc. Soc. exp. Biol. Med.*, **90**, 210.